# Accuracy of the product of symphysio-fundal height and abdominal girth in prediction of birth weight among term pregnant women at Keffi, Nigeria

**DOI:** 10.4102/phcfm.v12i1.2113

**Published:** 2020-06-10

**Authors:** Bolanle O. Ariyo, Stephen Yohanna, Jelil O. Odekunle

**Affiliations:** 1 Department of Family Medicine, Federal Medical Centre, Keffi, Nigeria; 2 Department of Family Medicine, Bingham University Teaching Hospital, Jos, Nigeria; 3 Statistics and Research Unit, Federal Medical Centre, Keffi, Nigeria

**Keywords:** Dare’s formula, birth weight prediction, symphysio-fundal height, abdominal girth, Keffi, Nigeria

## Abstract

**Background:**

A reliable prediction of foetal birth weight aids in decision regarding time and mode of delivery.

**Aim:**

This study aimed to determine the accuracy of the product of symphysio-fundal height and abdominal girth in predicting birth weight among pregnant women in Keffi, Nigeria.

**Setting:**

The study involved pregnant women presenting for delivery at the Federal Medical Centre, Keffi, Nigeria from July to October 2017.

**Methods:**

One hundred and fifty-three pregnant women at term with singleton foetuses were recruited by systematic random sampling. An interviewer-administered questionnaire was used to obtain their socio-demographic data, past medical and obstetric history. Symphysio-fundal height and abdominal girth measurements were used to calculate the estimated foetal weight. This was compared with the actual birth weight. Absolute percentage error was used to determine the overall predictive error of Dare’s formula. Data were analysed using SPSS version 20.0. Statistical significance was set at *p* < 0.05 and 95% confidence level.

**Results:**

The mean age of the participants was 29.65 ± 5.15 years. The mean gestational age was 39.5 ± 1.2 weeks. There was a significant correlation (*r* = 0.52, *p* < 0.001) between the estimated foetal weight and the actual birth weight. Ninety (66.2%) of the babies within normal weight and six (85.7%) of macrosomic babies were correctly predicted. None of the low birth weight babies was correctly predicted by the formula.

**Conclusion:**

Dare’s formula accurately predicted normal weight and macrosomic babies, but less accurately predicted low birth weight babies.

## Introduction

Birth weight is the first weight of the newborn obtained shortly after birth.^1^ It is a measure of foetal growth and a composite of many components, including bone, internal organs, muscle, fat and fluids. It is an important determinant of perinatal outcomes. The determinants of birth weight include gestational age at delivery, maternal race and other parental, environmental and pregnancy-specific factors.^2^ The normal birth weight ranges between 2500 g and 4000 g. Birth weights outside this range have been found to be associated with increased risks of perinatal morbidity and mortality.^3,4^

Prenatal estimation of foetal weight is an important aspect of birth preparedness. It aids in decision-making and preparations regarding the time and mode of delivery, perinatal care required and level of care or facility required. It is also useful for assessing prognosis and perinatal counselling on likelihood of survival of preterm and small for gestational age (SGA) babies.^3,4,5^

Foetal weight estimation can be done by biochemical, clinical and radiological means. None of the currently available techniques of foetal weight estimation is foolproof in terms of accuracy.^2,3^

The two most commonly used methods for predicting birth weight are the clinical and ultrasonographic methods.^2^ Clinical methods are less costly and can be performed by less experienced examiners. Ultrasonographic foetal weight estimation requires expensive equipment and trained personnel. The clinical method is a useful alternative in resource-poor settings.

Clinical methods of foetal weight estimation include tactile assessment of foetal size, clinical risk factor estimation, maternal self-estimated foetal weight (EFW) and birth weight prediction equations.^2^ The birth weight prediction formulas are based on symphysio-fundal height (SFH) measurements. These include Johnson’s, Ojwang’s, Dare’s and Dawns’ formulas amongst others. Some of these formulas are quite complex and difficult for routine use.^2^

Dare’s formula is a simple formula in which the product of abdominal girth (AG) and SFH is used to estimate foetal weight. It has been found to correlate well with actual birth weight (ABW) in previous studies.^6,7,8^ There is, however, paucity of studies on the accuracy of this formula in the authors’ region of practice. This study aimed to determine the accuracy of Dare’s formula in the prediction of birth weight amongst term pregnant women in the Federal Medical Centre (FMC), Keffi, Nigeria.

## Materials and methods

It was a cross-sectional analytical study conducted from 10 July to 31 October 2017 at the labour ward of the FMC, Keffi. Keffi is the Headquarters of Keffi Local Government Area of Nasarawa State, Nigeria and has an estimated population of 92 550 (2006 National census). An average of 200 new bookings and 500 return patients are seen monthly in the antenatal clinic, with an average of 120 deliveries per month.

All pregnant women who presented to the labour ward for delivery during the study period were eligible to participate in the study. The inclusion criteria were as follows:

- Booked pregnant women at term (gestational age 37 completed weeks to 41 weeks plus 6 days).
- Women whose gestational ages could be confirmed by last menstrual date or ultrasound dating at or before 20 weeks gestation.
- Patients with live singleton foetus in longitudinal lie with intact membranes.
- Patients in the first stage of spontaneous labour and those admitted for elective induction of labour or caesarean delivery.
- Patients who granted consent to participate in the study.

Comprehensive physical examination and ultrasound scan were conducted for all the women at the time of recruitment into the study. Those who had fibroid, ovarian tumour or cyst and poly or oligohydramnios were excluded from the study.

The minimum sample size was calculated using the Cochrane formula for determining the representative sample for proportions in a large population and the finite population correction for proportions recommended for adjusting the sample size for small study populations:^9^

$$\text{no}=\frac{Z^{2}\times p\times (1-p)}{e^{2}}$$ [Eqn 1]

where no is the minimum sample size required for a large population; *Z* is the 1.96 which is the standard normal deviate at 95% confidence interval level and *p* is the expected proportion of estimated birth weight correct to within 10% of ABW. This study used 69.5% from a similar study conducted by Njoku et al.^10^ in Calabar, Nigeria; *e* is the absolute precision level of 5%.

Hence:

$$\text{no}=\frac{1.96^{2}\times 0.695\times (1-0.695)}{0.05^{2}}$$ [Eqn 2]

$$\begin{array}{lll} \text{no} & = & \frac{1.96^{2}\times 0.695\times 0.305}{0.05^{2}}\\ \; & = & 325.7\;(\text{approximated to }326) \end{array}$$ [Eqn 3]

Because the population under study was about 240 women expected to deliver during the study period, the formula for finite population correction^9^ was applied to get the appropriate sample size as follows:

$$n=-\frac{\text{no}}{1+\frac{\left(\text{no}-1\right)}{N}}$$ [Eqn 4]

where *N* is the study population size and *n* is the adjusted minimum sample size:

$$n=\frac{326}{1+\frac{\left(326-1\right)}{240}}$$ [Eqn 5]

$$n=\frac{326}{1+\frac{\left(325\right)}{240}}=\frac{326}{2.35}=138.7\;(\text{approximated to }139)$$ [Eqn 6]

Thus, the minimum sample size required for the study was 139. An additional 10% of this (14) was added to allow for possible data loss. Therefore, 153 participants were recruited for the study.

The nature and purpose of the study were explained to prospective participants during group antenatal health education sessions. The participants were recruited through a systematic random sampling technique. An estimated 120 women were expected to deliver per month over the 8 weeks of the study, giving a sampling frame of 240 and a sampling interval of 2. The first participant was selected by random sampling through a ballot. Thereafter, every second eligible and consenting participant was selected until 153 women who fulfilled the inclusion criteria were recruited. Interviewer-administered questionnaires were used to obtain socio-demographic data and medical and obstetric history. Measurements of the SFH and AG were taken by the principal researcher or trained designated senior resident doctors using a flexible tape measure calibrated in centimetres. This was used to calculate the EFW in grams using the formula (EFW) in grams = SFH × AG in cm, where 1 cm = 1 g. The babies were weighed within 1 h of delivery by the principal researcher and trained midwives using an analogue neonatal weighing scale (Waymaster® England). The scale was frequently checked and adjusted for zero error.

Data were analysed using IBM SPSS Statistics for Windows, version 20.0. (IBM Corp., Armonk, NY, USA). The EFW was compared with the ABW using paired samples *t*-test. Accuracy of estimated birth weight was determined using percentage error calculated as [(ABW–EBW)/ABW] × 100. This provided information on whether Dare’s formula overestimated or underestimated the BW of babies. Also, absolute percentage error was used to determine the overall predictive error of Dare’s formula. Frequencies and percentages were used to determine accuracy within 10% of ABW. Statistical significance was set at *p* < 0.05 and 95% confidence interval level.

### Ethical consideration

Ethical clearance for the study was obtained from the Health Research Ethics Committee of the Federal Medical Centre Keffi (Approval no. FMC/KF/HREC/141/17). Participation in the study was voluntary and all participants provided written, informed consent at the time of recruitment.

## Results

The mean age of the participants was 29.65 ± 5.15 years. All the participants were married. About half of them (77; 50.3%) had secondary education. The majority of the participants (102; 66.7%) were employed. The mean estimated gestational age of the participants at delivery was 39.5 ± 1.2 weeks. The mean parity was 1.62 ± 1.50. Only a few of the women had co-morbidities that could have impacted negatively the birth weight. Fifteen (9.8%) participants had a history of high blood pressure, two (1.3%) had diabetes mellitus, three (2%) had anaemia, whilst nine (5.9%) and three (2%) had a history of delivery of large and small babies, respectively. The socio-demographic, obstetric and medical details of the participants are presented in Table 1.

**TABLE 1:** Socio-demographic, obstetric and medical details of participants.

Variables	Frequency	Percentage = 100 age group (years)
**Age group (years)**		
15–24	22	14.4
25–34	104	68.0
35–44	27	17.6
**Level of education**		
No formal education	6	3.9
Primary	8	5.2
Secondary	77	50.3
Tertiary	62	40.5
**Occupational status**		
Unemployed	51	33.3
Employed	102	66.7
**Religion**		
Christianity	115	75.2
Islam	38	24.8
**Marital status**		
Married	153	100
Parity		
0–2	88	57.5
3–4	60	39.2
5–6	3	2.0
7–8	2	1.3
**Abdominal girth**		
< 100	79	51.6
100 and above	74	48.4
**Pre-existing or current history of High Blood Pressure**		
Yes	15	9.8
No	138	90.2
**Pre-existing or current history of diabetes**		
Yes	2	1.3
No	151	98.7
**History of anaemia in current pregnancy**		
Yes	3	2.0
No	150	98.0
**History of delivery of large babies**		
Yes	9	5.9
No	144	94.1
**History of delivery of small babies**		
Yes	3	2.0
No	150	98.0
**Estimated gestational age at delivery**		
37–37 weeks 6 days	9	5.9
38–38 weeks 6 days	21	13.7
39–39 weeks 6 days	47	30.7
40–40 weeks 6 days	42	27.5
41–41 weeks 6 days	34	22.3
**Baby’s gender**		
Male	82	53.6
Female	71	46.4

*n* = 153.

Mean ± standard deviation = 29.65 ± 5.15.

**FIGURE 1:**
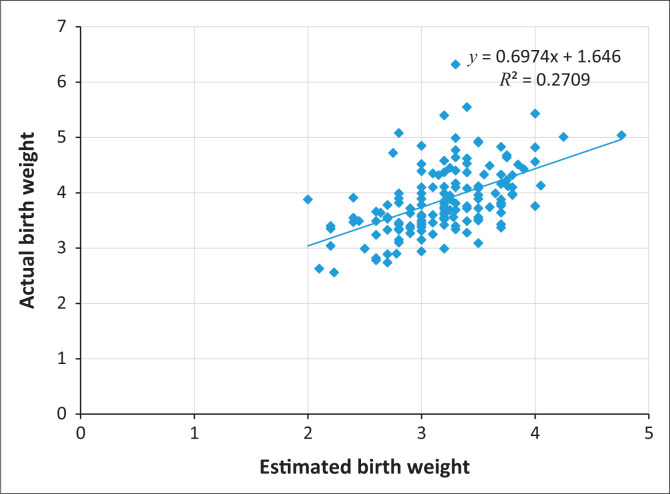
Scatter diagram comparing the estimated and actual Foetal weights.

The mean EFW was 3870 g ± 620 g. Majority of the babies, 136 (88.9%), were within the normal birth weight range of 2500 g – 4000 g. The mean ABW was 3190 g ± 460 g (Figure 1).

**FIGURE 2:**
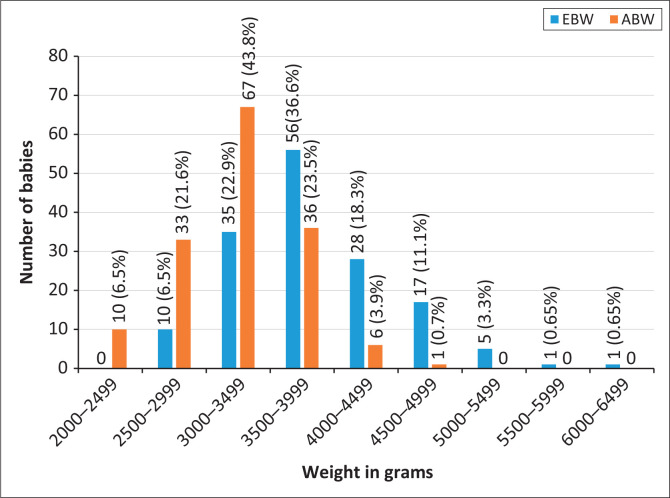
Bar chart comparing the estimated and actual birth weights of babies of the study participants.

Comparison of EFW and ABW showed that Dare’s formula underestimated the number of babies whose ABW was less than 3500 g, whilst it overestimated the babies whose ABW was 3500 g and above (Figure 2).

Only 37 (24.2%) of the study participants had their EFW correct to within 10% of the ABW. About 90 (66%) of the babies with birth weights within normal range and 6 (86%) of macrosomic babies were correctly predicted. None of the low birth weight babies was correctly predicted by the formula. The EFW using the Dare’s formula correlated best with the ABW within the 3500 g – 3999 g range. There was a positive and significant correlation (*r* = 0.5, *p* ≤ 0.001) between the EFW and ABW. There was significant difference in the means of EFW and ABW.

## Discussion

In this study, Dare’s formula (product of SFH and AG) was used to predict birth weight amongst term pregnant women in FMC, Keffi, Nigeria. The mean age of the participants in this study was comparable to what was found in similar studies conducted elsewhere in Nigeria,^8,10^ in agreement with the national (Nigeria) mean maternal age at childbirth which is 29.8 years.^11^ The mean parity of the participants in this study (1.6) was comparable to findings in other studies. Malik et al.^12^ in Islamabad had a mean parity of 1.66 ± 1.6 amongst their study participants. Torloni et al.^6^ in Brazil had a mean parity of 1.2 ± 1.5 amongst their study participants. The contrary was the case in Kano, North-Western Nigeria, where Ugwa et al.^13^ had a parity of 3 ± 2 amongst their study participants. This may be because of low level of education, early marriages and higher births amongst women in the North-Western region of Nigeria compared to Keffi in North-Central Nigeria.^14^ Female education has been found to increase the uptake of contraceptives with subsequent reduction in the rate of childbirth.^15^ The higher percentage of educated participants in this study might be responsible for the lower mean parity.

In this study, the correlation factor between EFW and the ABW was 0.52. This was lower than what was found in the study by Sharma et al.^16^ in India. Their study involved 303 pregnant women of any parity and age with a period of gestation greater than 28 weeks and in any stage of labour. They found a stronger correlation (0.75) between EFW and ABW. The reason for this may be the exclusion of obese patients from their study. Thombarapu and Agrawal^17^ in their study in India also had a higher correlation (0.726) between EFW and ABW using the Dare’s formula. Ugwu et al.^8^ in Enugu, Nigeria, also had a stronger correlation (0.71) between the EFWs and ABWs amongst their study participants. This may also be a result of the exclusion of women that weighed greater than 95 kg from their study. Weight of the participants was not considered in this study in Keffi. This may explain the lower correlation between the EFWs and ABWs in this study.

Torloni et al.^6^ in a study conducted in Brazil compared the accuracy of Dare’s formula, Johnson’s formula and mother’s assessment of foetal weight and ultrasound in prediction of birth weight. They analysed the results obtained from 100 women with full-term, cephalic, singleton pregnancies who delivered within 3 days of the foetal weight estimation. They found that birth weights were correctly estimated to within 10% of the actual in 59%, 57%, 61% and 65% of the participants using the mother’s estimate, Dare’s formula, Johnson’s formula and ultrasound estimate, respectively. They postulated that Dare’s formula was less accurate than Johnson’s formula for prediction of birth weight because of the lack of correction for obesity in the Dare’s formula. Twenty-four per cent of their participants were obese. Thus, they recommended a larger study to test the accuracy of the Dare’s formula in obese women.^6^ Another study conducted by Deeluea et al.^18^ in Thailand showed differences in the fundal height growth curves of underweight, overweight and obese pregnant women compared to those with normal weights. It can be extrapolated from this study that the weight of the woman influences the SFH and by extension of the EFW because the SFH is one of the variables used to calculate the EFW in the Dare’s formula.

The correlation in this study was, however, comparable to what was found by Mortazavi and Akaberi^7^ in Iran. They found a correlation of 0.56 between the EFW and the ABW amongst their study participants. It was also deduced from their study that the Dare’s formula will predict foetal macrosomia at a cut-off point of 3900 g and low birth weight at a cut-off point of 3000 g. This implied a relative underestimation of large birth weight babies and overestimation of the weight of low birth weight babies.^7^ This was contrary to the findings in the present study, where the formula was found to underestimate weights below 3500 g and overestimate birth weights from 3500 g and above. Mortazavi and Akaberi evaluated 795 participants in their study. This was a larger study compared to the present study. They also utilised a non-random sampling technique in recruiting their participants thus giving room for bias. They also excluded women who weighed greater than 91 kg which would have screened out women who are likely to have large birth weight babies.

Unlike the finding in this study, Sharma et al.^16^ found that there was no significant difference between the mean EFW obtained from the product of SFH and AG and the mean ABW in their study participants. The exclusion of obese women from their study might have given them better results. Their study participants were also not limited to those with term pregnancies. They included women that reported in labour from greater than or equal to 28 weeks gestation.

The proportion of the participants who had EFW correct to within 10% of ABW in this study was quite low (24.2%) compared to 81% found amongst the participants of the study by Yadav et al.^19^ in India. The study by Yadav et al. was carried out over a longer duration (18 months) than the present study and they had a larger number of participants (200). This might have been responsible for the differences in the results obtained. The racial differences might have also played a role.

Esmaeilou and Mohammadi^20^ in Iran also had a higher proportion of their participants (68%) with EFW within 10% of the ABW. They, however, had a smaller sample size with 98 participants. Torloni et al.,^6^ despite not excluding obese mothers from their study in Brazil, had a higher proportion of their participants (57%) with EFW (predicted from Dare’s formula) within 10% of ABWs. It was, however, observed that their sampling technique was biased as the women who reported to the study centre during the period of their study did not have an equal chance of being selected to participate in the study. They also had a smaller sample size (100) compared to the present study.

The percentage of the participants that had EFW correct to within 10% of the ABW in the study by Ugwu et al.^8^ in Enugu, Nigeria was comparable to this study. They had 35% of the EFWs for their participants within 10% of ABW. They compared the accuracy of ultrasonic method of foetal weight estimation and the clinical method using Dare’s formula. They found that both methods overestimated birth weight with the error more with the clinical method (Dare’s formula). It was noted that the participants of the present study and that of Ugwu et al. in Enugu had similar characteristics. The mean maternal age, parity and mean gestational age were similar in both studies. This probably accounted for the similarity of the findings of both studies.

Malik et al.^21^ in India found that Dare’s formula overestimated birth weight in 84% of the participants in their study. This was higher than was found in the present study, where Dare’s formula overestimated birth weight in 33.8% of babies with normal weight. Their study was, however, a non-randomized study which was undertaken over a longer duration of 14 months. Their study participants included those with gestational period greater than 34 weeks and obese women were excluded from their study. Hence, the disparity in the findings of the two studies.

## Conclusion

About 24% of the study participants had their EFW correct to within 10% of the ABW in this study. The EFW correlated well with the ABW with a correlation factor of 0.52. The findings of this study showed that the Dare’s formula was fairly accurate in the prediction of normal weight (66.2%) and macrosomic babies (85.7%). The formula failed to accurately predict low birth weight babies. This suggests that its use in this environment may be limited in case of suspected low birth weight. Dare’s formula should be combined with other methods of foetal weight estimation to make decisions on the management of such patients.

Further research to compare the accuracy of birth weight prediction with Dare’s formula in obese and non-obese mothers is recommended.
